# Impact of COVID-19 pandemic on families of children and adolescents with diabetes: maternal perspective

**DOI:** 10.1590/0034-7167-2025-0122

**Published:** 2026-07-27

**Authors:** Milena de Lucca, Diene Monique Carlos, Ana Carolina Andrade Biaggi Leite, Giovanna Cristina Machado Kayzuka, Rebecca Ortiz La Banca Barber, Tatiane Geralda André, Paula Saud De Bortoli, Lucila Castanheira Nascimento

**Affiliations:** I Universidade de São Paulo. Ribeirão Preto, São Paulo, Brazil; II Universidad Pública de Navarra. Pamplona, Navarra, Spain; III Children’s Hospital Los Angeles. Los Angeles, California, United States of America

**Keywords:** COVID-19, Diabetes *Mellitus*, Type 1, Adolescent, Child, Family Nursing., COVID-19, Diabetes *Mellitus* Tipo1, Adolescente, Niño, Enfermería de la Familia.

## Abstract

**Objectives:**

to investigate maternal perspectives on the impacts of COVID-19 pandemic the daily lives and diabetes care of families of children and adolescents with type 1 diabetes.

**Methods:**

qualitative study guided by Morin’s complexity paradigm. Remote, audio-recorded, semi-structured interviews were conducted with eight Brazilian mothers recruited through social media. Data were examined using inductive content analysis.

**Results:**

three categories emerged: a) Intensifying care: between the fear of the unpredictable and infection-prevention routines; b) (Re)assuming control: maternal responsibilities over the disorder caused by the pandemic; and c) Reinventing family life: actions grounded in supportive care.

**Final Considerations:**

maternal perspectives reveal multidimensional pandemic-related impacts on family routines and diabetes care and highlight challenges and coping strategies adopted. The findings can inform emotional support interventions and initiatives to strengthen care for these families.

## RESUMEN

**Objetivos:**

analizar las perspectivas maternas sobre las repercusiones de la pandemia por COVID-19 en la vida cotidiana de familias de niños y adolescentes con diabetes mellitus tipo 1.

**Métodos:**

estudio cualitativo, fundamentado en el paradigma complejo, con entrevistas semiestructuradas, remotas y audiograbadas a ocho madres reclutadas mediante redes sociales; los datos se sometieron a análisis de contenido inductivo.

**Resultados:**

emergieron tres categorías: a) Redoblando los cuidados: entre el miedo a lo imprevisible y los rituales de prevención del contagio; b) (Re)asumiendo el control: responsabilidades maternas ante el desorden instaurado por la pandemia; y c) Reinventando la convivencia familiar: acciones basadas en el cuidado apoyado.

**Consideraciones Finales:**

las perspectivas maternas revelan impactos multidimensionales de la pandemia en el cuidado infantil, señalan sobrecarga y adaptaciones familiares y pueden orientar intervenciones de apoyo emocional y fortalecimiento del cuidado a estas familias.

## INTRODUCTION

On March 11, 2020, the WHO declared a public health emergency because of the rise in COVID-19 cases, which led to the adoption of restrictions that affected the population^(1)^. Some of these measures caused significant impacts on the economy, health, education, and social relationships, mainly because of the need for social distancing; changes in daily routines, such as the suspension of in-person school, sports, and leisure activities; the adoption of strict hygiene and care protocols; and the postponement of elective medical appointments and procedures^(2)^.

Among the population groups that were more vulnerable and more affected during the pandemic were children and adolescents, mainly because of their fragility and dependence. In addition, beyond the 0-19-year age range considered by the WHO, these are individuals who are in an ongoing process of growth and development and whose specificities should be highlighted^(3,4)^. The changes in the learning environment brought about by the transition to remote teaching required adaptation to a new routine and reduced their interaction with peers and opportunities for recreational activities. This, in turn, markedly affected how these families were organized and how they functioned^(3)^.

When a chronic condition was present, even more evident vulnerabilities were identified, mainly because health services were limiting visits and because access to care became more difficult^(4)^. Among the most common chronic diseases in this age group is type 1 diabetes (T1D). In 2022, an estimated 1,520,000 children and adolescents younger than 20 years were living with T1D worldwide. The incidence of the disease has been increasing globally, with nearly 200,000 new T1D cases in the child and adolescent population in 2022 compared with 2021^(5)^. Brazilian data also deserve attention, since they show nearly 112,240 prevalent cases of T1D among children and adolescents in 2022^(5)^.

Besides being an important public health problem, T1D entails several changes for pediatric patients and their families, since from the time of diagnosis, they must adapt to new routines and habits to ensure effective treatment and maintain quality of life, such as daily insulin administration, metabolic control, an adequate diet, and regular physical activity^(6)^. In this context, it becomes important to understand how these families deal with such demands, readjust their roles, and often perform highly complex tasks without prior training or skills^(4)^.

This observation is particularly relevant because the available studies on this topic have mainly focused on the physical and mental health of this population group, highlighting a knowledge gap regarding the impacts of the COVID-19 pandemic on family dynamics^(7-10)^. Recent studies have reiterated the need to broaden the understanding of COVID-19-related impacts on families of children with chronic conditions, addressing their shortand long-term repercussions from a complexity perspective, that is, considering the relationships among the multiple elements that make up a phenomenon and their contextual interactions^(11-14)^. Moreover, the characteristics and consequences of COVID-19 for children and adolescents across the spheres of health, education, and development should be further investigated^(12,13)^.

When the family is viewed in a broader sense, as a system in which members mutually influence one another and adapt to adversity dynamically^(15)^, the central role of mothers in caring for children with chronic conditions becomes evident. The literature shows that, in most situations, they assume primary responsibility for treatment management, coordination with health care services, and the child’s emotional support^(16)^. In this regard, the burden intensified during the pandemic justifies the maternal focus adopted in this study, which led to the following research question: What are the COVID-19 pandemic-related impacts on the daily lives of Brazilian families of children and adolescents with T1D, from the mothers’ perspective?

## OBJECTIVES

To investigate maternal perspectives on COVID-19 pandemic-related impacts on the daily lives of families of children and adolescents with type 1 diabetes.

## METHODS

### Ethical aspects

In accordance with Brazilian National Health Council Resolution 466/2012, the Research Ethics Committee of the proposing institution approved the study in 2022. After receiving clear and objective information about the study, the mothers who agreed to participate signed the Informed Consent Form (ICF) digitally via Google Forms and then received an electronic copy. They could also request a signed copy from the researchers, which would be sent to their email addresses. Throughout data collection and analysis, participants’ anonymity was preserved, and the interviews were identified numerically (e.g., INT1, INT2, and so on).

### Study type and theoretical framework

To ensure the rigor of this study, its description followed the recommendations of the Consolidated criteria for reporting qualitative research (COREQ)^(17)^, with a detailed presentation of the method. A rigorous data analysis was also carried out by an experienced research team, which ensured the study’s trustworthiness and credibility. In addition, transferability and confirmability were supported by presenting the participants’ characteristics and by describing the study’s limitations and strengths.

A qualitative study was carried out, grounded in Morin’s complexity paradigm. This framework, whose foremost proponent is Edgar Morin, offers a worldview that challenges conventional ways of conceiving knowledge production from epistemological, psychosocial-anthropological, and ethical perspectives. The term “complex” comes from the Latin “complexus”, meaning “woven together”. Thus, it considers opposing elements present in the same phenomenon from a dialogical perspective, embracing non-linearity, disorder, and uncertainty. It seeks not to deny, but to go beyond the responses and possibilities of simplifying thinking^(14)^. It incorporates a systemic perspective, which links knowledge of the parts to the whole, and a hologrammatic perspective, which considers the relationships among the parts and the presence of the whole in those same parts. This choice not only welcomes the systemic view of the family^(15)^ adopted by the authors, but it also considers the interweaving between the parts (the child or adolescent and family members) and between these parts and the whole (living with T1D and the pandemic). It also assumes that the different elements of a given phenomenon remain in constant interaction, from which singular properties emerge. These undergo self-eco-organization, that is, movements of disequilibrium and re-equilibrium in a constant search for organization, while being affected by the environment. This last aspect underpinned the authors’ interest in further examining the COVID-19 pandemic-related impacts on the daily lives of the families studied.

### Setting and data source

The study was conducted during the COVID-19 pandemic, when social distancing measures restricted in-person meetings. For this reason, data collection was conducted remotely, via individually scheduled interviews on Google Meet and Zoom. Mothers were included if they met the following criteria: being the mother of a child or adolescent aged 7 to under 18 years, with a medical diagnosis of T1D. Mothers younger than 18 years, as well as those who, for any reason, were not the primary caregivers of their children in daily life during the pandemic period, were excluded.

### Methodological procedures

Participants were selected by purposive and convenience sampling and were recruited using snowball sampling^(18)^. The first author, a nurse and doctoral student with experience in qualitative research, collected the data under the supervision of the advisor (last author). The researcher had no prior contact with the participants. Initial recruitment took place by posting the study on social media (Instagram and WhatsApp) via the authors’ personal accounts, which were already used for this type of recruitment. Posts (folders) containing the study topic and objective were published. Because the posts were public, the information spread and reached people interested in the topic. Mothers who viewed the posts expressed their interest by contacting the lead researcher via Instagram or WhatsApp. A time was then scheduled to provide further information and, if they agreed to participate, informed consent was obtained by signing the Informed Consent Form. Mothers who were included were asked to indicate other potential participants, and this process continued according to snowball sampling^(18)^. The viral reach of social media facilitated rapid dissemination of the study, overcoming geographic barriers and connecting people with shared interests who otherwise would have been difficult to reach.

The total number of participants was determined when the researchers, through progressive analysis conducted alongside data collection, found that the data set was sufficient to meet the study objective^(19)^. Data saturation was identified when the narratives began to show recurring patterns and no new meaningful perspectives emerged, indicating that the material already encompassed the diversity and depth of the phenomenon under investigation. This decision was guided by core principles of qualitative research, which emphasize the quality and depth of the information collected rather than on the number of participants^(19)^. In qualitative studies, the priority is to explore participants’ experiences and perspectives in depth, often yielding rich, detailed material. When no new themes or relevant perspectives emerged during analysis, data collection was ended.

### Data collection and organization

A single interview was conducted with each mother between January and March 2023. Before the interviews, the lead researcher collected sociodemographic and clinical data using a form previously developed by the research team, which included questions on the mother’s age, sex, educational attainment, and city of residence, as well as the child’s/adolescent’s city of residence and date of T1D diagnosis.

The semi-structured interviews followed a guide with guiding questions to explore: management of the chronic condition and its care; the impact of the pandemic on school and family routines; the experience of school dropout caused by social distancing measures; and school reentry after restrictions were eased. Because the World Health Organization officially declared the end of the COVID-19 pandemic in May 2023, collecting data close to this time frame was a strategic decision. It avoided an excessive time gap, which could lead families to forget relevant aspects, and, at the same time, prevented interviewing them too close to the event, which could trigger intense emotions and distress.

The interviews were remotely audio-recorded and fully transcribed, with confidentiality and anonymity ensured throughout. They were individual interviews and lasted, on average, 75 minutes, ranging from 30 to 165 minutes.

### Data analysis

Inductive content analysis of the transcribed interviews was carried out in three phases: (I) preparation, (II) organization, and (III) reporting of results^(20)^.

In the first phase, the transcripts were read repeatedly to obtain a comprehensive understanding of the material and to identify meaning units related to the study theme^(20)^.

In the second phase, the material was coded and categorized. From the meaning units, we identified codes that reflected aspects of the content, such as: anxiety and non-acceptance; fear of COVID-19 infection; depressive processes; maternal feeling of powerlessness; seeking professional help; and support from the extended family, among others. These codes were then grouped into subcategories and categories, and a general description was formulated for each of them to provide a more in-depth presentation of the phenomena that addressed the study objective.

In the third phase, the categories were named according to their characteristics and articulated with the complexity paradigm, and they are presented in the Results section^(20)^. All phases were conducted by the first and last authors and were discussed with the entire research team. The researchers either had experience with this type of data analysis or were specialists in the topic.

## RESULTS

Eight mothers of children and adolescents with T1D living in six cities in the states of São Paulo, Minas Gerais, and Pará were interviewed. Maternal age ranged from 33 to 50 years, with a median of 40 years. As for education, 87.5% had completed higher education, and 12.5% had completed high school.

The children and adolescents with T1D were aged 8 to 17 years, with a median age of 11 years. Most were male (62.5%). Time since T1D diagnosis ranged from 0.9 to 7 years.

From the analytic process, three categories were developed, each with its respective subcategories: a) Intensifying care: between the fear of the unpredictable and infection-prevention routines; b) (Re)assuming control: maternal responsibilities over the disorder brought about by the pandemic; and c) Reinventing family life: actions grounded in supportive care.

### a) Intensifying care: between the fear of the unpredictable and infection-prevention routines

#### Care routines

This category examines how mothers understood the pandemic and how they turned preventive measures into care routines. According to the participants, infection-prevention practices had to be intensified for children and adolescents with T1D:

*Extra care, because we already had to be careful and, since it was the pandemic, we had to be extra careful with everything.* (INT3)

Hygiene practices such as using hand sanitizer, washing hands, and changing clothes right after arriving home became part of everyday care and reflected the broader pandemic scenario in the routine of caring for a child with T1D:

*I’d get home and right away wash the clothes, spray alcohol on my body, wash my hands, and it became that thing, right? Cleaning everything so he wouldn’t get* [COVID-19]. (INT1)

Within this pandemic context affecting family routines, mothers also stressed the need to maintain social distancing and to keep children away from contact with others:

*We cut all social activities, church, everything. She didn’t even go to the supermarket; we really cut everything.* (INT7)

To achieve this, some families adopted strategies such as temporarily moving to a place farther from the community:

*We have a house by the reservoir, so when my husband started working from home I said, “Let’s go there, because it’s farther away”.* (INT8)

A constant concern about not letting their children leave the house also emerged:

*We tried not to leave the house at all, especially her.* (INT7)

*You could tell he had already internalized the danger of having diabetes and getting* [COVID-19] […] *he said, “No, I don’t even want to go out, I don’t want to see anyone”. All because of that.* (INT8)

#### Maternal concerns: the fear that took hold

In the pandemic context, and because they understood their children as being at higher risk for severe COVID-19, the mothers reported a persistent, underlying fear that their children would become infected. This feeling led many of them to report a heavy mental burden, as well as depressive symptoms, distress, crying episodes, decreased appetite, weight loss, frustration, nonacceptance, and a sense of powerlessness in the face of what they were experiencing:

*During the pandemic, there was frustration, there was fear, there was distress, there was a bit of despair.* (INT1)

*I lost weight; I didn’t even feel like eating, I kept having that fear, that panic about him getting* [COVID-19]. (INT8)

One mother said she was terrified that insulin production would stop worldwide, since all efforts were focused on producing the COVID-19 vaccine, which clearly showed the unpredictability brought about by the pandemic:

*We were in such a difficult situation, everybody focused on the vaccine. And if insulin production stopped, what would we do? I felt very insecure because of that unpredictability. When you have a child with a chronic condition, what you value most is having the medication always at hand, because that’s their life, right?* (INT8)

### b) (Re)assuming control: maternal responsibilities over the disorder brought about by the pandemic

#### Life put on hold

This category highlighted the mothers’ efforts to (re)organize family life in response to the disorder brought about by the pandemic, showing how changes in the family as a whole were reflected in each member’s routine and, in turn, reshaped the family dynamic. The period of social distancing and suspension of daily activities was experienced as if life had stopped, and planning was no longer possible:

*And during the pandemic, when everything stopped, our life was just put on hold; it felt like there was no planning anymore. We were stuck in that situation. We had to wait to see what would happen.* (INT2)

#### Taking care of themselves: seeking balance

The participants also reported trying to maintain control over care coordination, taking the lead in contacting health care services, recognizing signs and symptoms of T1D worsening, and organizing the family routine, in a self-organizing movement to support their children’s and family’s care. In this period of overload, some of them, after acknowledging their own vulnerabilities and needs, sought professional help and started psychiatric medication and psychotherapy:

*I had to stay in control, I needed psychological treatment, I realized I needed it* […] *I need to show determination and strength, but I’m not made of iron. I need help too.* (INT2)

#### Support networks

Faced with the fear that had taken hold, the primary sources of support for coping were the support network formed by the health care providers who followed their children, the extended family, the group of mothers of children with T1D, and faith:

*At first, I was scared, but since I really trust the doctors who follow him, they reassured me a lot, you know? So it went away quickly.* (INT4)

*At the beginning, the ones who were essential to me were my father and his wife. They live near my house and they came every day to help me; if it hadn’t been for them, I wouldn’t have managed, because I cried out of despair, of not being able to do everything, to handle everything.* (INT3)

*Several mothers going through the same situation. That is very important, one supporting the other. Everything one mom learns from studying, she passes on to the others. Every day, it’s diabetes knowledge, every day. That is very important.* (INT6)

*I believe faith was what sustained us the most. It was the foundation of everything.* (INT2)

### c) Reinventing family life: actions grounded in supportive care

#### Reorganizing daily routines

Given the pandemic context and the introduction of social distancing, the mothers had to reorganize daily tasks and care routines for their children with T1D. There was a need to adjust the whole family’s diet, since the children started spending more time at home and having more meals together:

*I was here at home with her the whole time, always explaining, monitoring, and we also changed our diet because of her - we didn’t buy things just for us and not for her* […] *being at home helped.* (INT7)

Unlike the period before the pandemic, when mothers did not stay at home for long periods, this new circumstance - marked by being at home most of the time - made it possible for them to monitor their children’s eating more closely, which favored better adherence to the dietary plan:

*He gets really hungry, so he wanted to eat all the time, but because I was at home it was easier for me to control it* […] *I tried to keep it under control, but if you turn your back, they eat - they eat on their own.* (INT6)

Carbohydrate counting also helped maintain good glycemic control during this period:

*That’s how we managed, you know? We tried to do the carbohydrate counting so it wouldn’t throw him off so much.* (INT2)

*It was actually a time when his blood glucose went down a lot. During the pandemic, he didn’t have any really high spikes.* (INT1)

Children and adolescents had to stop participating in sports at clubs and schools. As an alternative, families started to do them at home, with functional workouts, running, jumping rope, playing ball in the yard, and dancing:

*We’d do it here at home. We did these functional workouts, we ran here* […] *sometimes he didn’t want to, but he had to - run in the garage, jump rope, do something.* (INT6)

*He was always out front playing ball, without getting together with other kids.* (INT4)

*Because of the pandemic, we had to adapt. She likes to dance too, so I put some music on. Since I’m overweight, I took the opportunity to dance with her - that’s how we did it.* (INT7)

Another adaptation during this period was starting medical or nutritional appointments through online platforms, which some mothers viewed as insufficient:

*Then came the online appointments, and online appointments aren’t the same, the doctor needs to see him. I never did an online appointment with his doctor; I took him there. I did one with the nutritionist because I was afraid. My God, how is he not going to see my son? How is that going to work?* (INT8)

#### Resuming play and close family interaction

Because they had to spend more time at home, the mothers also looked for ways to keep the children engaged, such as playing together, card games, cooking/trying out recipes, and question-and-answer games:

*I tried to find more ways for us to play together* […] *we played a question game and I put a little plate with flour on the table and whoever got the question wrong* […] *it was like a pie in the face, you know?* (INT2)

This made it possible for families to spend more time together and, in doing so, increased interaction among family members:

*What I most needed to change, what I noticed, was really the interaction part, dealing with things at home so he wouldn’t suffer so much from the distancing everyone was going through.* (INT2)

Children who had siblings at home were able to enjoy their company, playing, gaming, and watching series together:

*Fortunately, he has a brother; he plays with him, watches series, those things that helped.* (INT8)

#### Interruptions in ongoing processes and return to activities

Mothers reported that adolescents, in particular, experienced anxiety and started reflecting on their diagnosis, facing moments of nonacceptance. The following excerpt illustrates the experience of an adolescent who felt that everything he was starting to do had been interrupted, since he was about to change schools, and the pandemic made it harder to make new friends and to experience an important stage of his development:

*We could tell he was anxious, and then he hit that point of not accepting it. Like: “I can’t believe I have diabetes!”.* (INT1)

As social distancing measures began to be relaxed, some mothers experienced their children’s school return in a dialogical way - they wanted them to go back to school and to social life, but there was still fear and insecurity:

*I held her back a little; I discussed it with the school because we had to reorganize our whole family routine so we could support her return to school.* (INT3)

*I had to put on those rose-colored glasses and be sure she would come home safe and sound.* (INT5)

## DISCUSSION

The COVID-19 pandemic affected the daily lives of children and adolescents with T1D and their families. In this context, infection-prevention practices were incorporated into family routines, with knowledge and actions for the child’s diabetes care being constantly (re)introduced. There was maternal self-eco-organization to (re)assume control, articulating the order-disorder-organization brought about by the pandemic in a search for balance, including for themselves, so they could organize care. These dynamics were evident in the categories “(Re)assuming control” and “Reinventing family life”. Faith, support from health care providers, the extended family, and peer support groups underpinned these efforts toward reorganization.

Caring for a child with T1D entails significant changes in family routines and recurrent concerns about managing and treating the condition^(6)^. During the COVID-19 pandemic, the higher risk of severe complications among people with comorbidities intensified the psychological distress of mothers caring for children and adolescents with T1D, as shown in this study and confirmed in the literature^(9,10,21-23)^. In addition, caregivers who were already experiencing anxiety and depressive symptoms reported worsening of these conditions during the pandemic^(24)^.

One study that sought to measure this impact assessed the psychological effects of the pandemic on caregivers of children and adolescents with T1D, comparing them with caregivers of children without diabetes^(25)^. Those caring for children with T1D were 60% more likely to experience worry and personal overload and were twice as likely to be concerned about their children’s care^(25)^. Similar concerns were described in other studies, such as fear of infection^(22)^, worries about reduced immunity and glycemic fluctuations^(21)^, and apprehension about a possible shortage of insulin and medical supplies^(21,22)^. This heightened apprehension led mothers to develop the care routines described in the Results section.

Spending longer periods at home also changed the daily routines of children and adolescents with T1D. Mothers reported closer monitoring of diet, blood glucose, and treatment adherence, as well as the need to adapt physical activity to the home setting. Although this scenario intensified disease surveillance, it required readjustments in family dynamics to meet the demands of children and adolescents. This control was crucial because during the pandemic, 60% of children and adolescents with T1D reported worse eating habits^(22)^, and 72% stopped regular sports practice^(9)^. In our study, mothers not only tried to control food intake but also sought to lessen the negative effects of interrupted physical activity by encouraging daily, joint exercise at home.

(Re)assuming control involved creative and systemic actions, such as adapted play, mostly led by the mothers. The autonomy-dependence dialogic relationship marked the return to in-person activities, particularly school-related ones, among children and adolescents with T1D. Mothers tried to foster autonomy in this population, while at the same time, they experienced fear because of the unpredictable elements of the pandemic context, which increased children’s and adolescents’ perceived dependence on maternal and family care.

The need for control affected diabetes management. During the isolation period, there was a 10% improvement in time in range (TIR), as well as a significant reduction in episodes of hypoglycemia and hyperglycemia among adolescents with T1D^(26)^. There is consistent evidence that people with T1D who remained at home during lockdown achieved better glycemic control^(27,28)^. These findings align with ours and show families’ capacity for self-eco-organization and adaptive self-regulation in the face of pandemic-related disruption.

However, exercising such close control during the pandemic created tension for mothers. Although it reinforced the importance of parental involvement in T1D management, it also increased the caregiver burden and strained family interactions, leading to frustration when control was lost^(29)^. The need for precision in daily diabetes care led parents to feel guilty when glycemic control did not meet expectations^(9)^. Even before the pandemic, they already experienced a significant emotional impact because of the demands of managing their children’s diabetes^(30)^, and the pandemic heightened this burden, sometimes resulting in adequate glycemic control but at a high emotional cost for parents^(9)^. From a complexity perspective, this reinforces the need to understand care in an unpredictable, non-linear context and to plan interventions by health care providers that consider order-disorder-organization dynamics, both for the child and for the family.

The literature has already described how difficult it is for these parents to entrust care to others, such as teachers, which results in a constant burden of vigilance over children and adolescents with T1D^(30)^. In our study, this contributed to feelings of insecurity about returning to in-person school activities. Trust in the diabetes care team and in family members, on the other hand, played a key role in maintaining glycemic control and establishing a consistent family routine^(23,29)^. During the COVID-19 pandemic, social support and telemedicine visits were important in reducing the psychological distress experienced by these families^(24)^, although adaptations were needed to ensure continuity of care, such as learning to use digital technologies^(23)^. Still, mothers in this study reported difficulty accepting the effectiveness of online visits and, similar to findings from other caregivers, expressed a preference for in-person appointments^(9)^.

The complexity, evidenced by emotional instability and abrupt changes in routine, can make it harder to recognize coping strategies and positive progress^(15)^. For this reason, it is essential to care for the caregiver as well. The long duration of the interviews in this study highlights the mothers’ need for a space to talk about their experiences and concerns. The range of narratives shows how important it is to more deeply explore these experiences in future research. Understanding their challenges and needs is essential to providing psychosocial support and self-care resources. By acknowledging and supporting mothers in this context, it is possible to promote mothers’ well-being and that of children with T1D, contributing to better quality of life and improved health outcomes. Despite the large number of studies on the consequences of the pandemic, there is still a gap in the literature regarding lessons learned from caring for children and adolescents with chronic conditions during this challenging period.

### Limitations of the study

The strengths of this study include collecting data after social distancing measures were lifted, which allowed mothers to share their experiences of returning to activities. Using technological tools for remote qualitative data collection enabled access to participants living in different regions. In addition, conducting a qualitative study and following the COREQ checklist ensured rigor in the description of the methods and findings.

A possible limitation is the educational attainment of the mothers interviewed, all of whom had completed higher education (n = 7) or were attending higher education (n = 1), which does not represent the reality of all families of children and adolescents with T1D, since the pandemic more severely affected socially and economically vulnerable families^(24,31)^. Moreover, although remote data collection was useful, it limited access to economically disadvantaged groups, whose experiences may have been different. The evidence presented here describes the specific characteristics of the families of children and adolescents with T1D included in this study. Therefore, transferability and interpretation of the findings to other contexts, such as other pediatric chronic conditions, should be done cautiously and always considering the participants’ contextual characteristics.

### Contributions to the field of nursing, health, or public policy

The maternal perspective, analyzed through a qualitative lens, offers a new contribution to the literature and to clinical practice, centered on lived experience and on the complexity involved, since the main studies published on the impacts of the COVID-19 pandemic are quantitative and, for the most part, provide only numerical data on glycemic control in children and adolescents with T1D. It is essential to recognize and understand how maternal burden increased during the pandemic so that health care providers can develop emotional support resources for caregivers and offer support in caring for children and adolescents, particularly during periods of heightened stress, such as pandemic and post-pandemic scenarios. In this regard, nursing has a central role in the longitudinal follow-up of children and adolescents with T1D, not only in clinical monitoring and glycemic control, but also in user embracement, qualified listening, and ongoing guidance for families. This involvement can improve care coordination and the quality of life of families living with T1D in childhood and adolescence.

## FINAL CONSIDERATIONS

This study investigated maternal perspectives on COVID-19 pandemic-related impacts on the daily lives of families of children and adolescents with T1D. Faced with the disorder brought about by the pandemic, families adopted preventive measures and routine changes to protect against the virus and to prevent T1D-related complications. The effort to (re)organize family life, especially when resuming activities after social distancing, led mothers to (re)introduce knowledge and creative, supportive actions to sustain their children’s care.

## Data Availability

The research data are available within the article.
