# Supplementary material for: Impact of COVID-19 pandemic on families of children and adolescents with diabetes: maternal perspective
Source: Rev Bras Enferm. 2026 Jul 27;79:e20250122. doi: 10.1590/0034-7167-2025-0122 (PMC13411322; doi:10.1590/0034-7167-2025-0122)
Supplement: Translation

# Translation: Repercussões da pandemia de COVID-19 para famílias de crianças e adolescentes com diabetes: perspectivas maternas

**DOI:** 10.1590/0034-7167-2025-0122pt

**Authors:** Milena de Lucca, Diene Monique Carlos, Ana Carolina Andrade Biaggi Leite, Giovanna Cristina Machado Kayzuka, Rebecca Ortiz La Banca Barber, Tatiane Geralda André, Paula Saud De Bortoli, Lucila Castanheira Nascimento

**Affiliations:** I Universidade de São Paulo. Ribeirão Preto, São Paulo, Brasil; II Universidad Pública de Navarra. Pamplona, Navarra, Espanha; III Children’s Hospital Los Angeles. Los Angeles, Califórnia, Estados Unidos da América

**Keywords:** COVID-19, Diabetes *Mellitus* Tipo1, Adolescente, Criança, Enfermagem familiar.

## Abstract

**Objetivos:**

investigar as perspectivas maternas a respeito das repercussões da pandemia de COVID-19 no cotidiano de famílias de crianças e adolescentes com diabetes mellitus tipo 1.

**Métodos:**

estudo qualitativo, fundamentado no referencial teórico do Paradigma Complexo. Realizaram-se entrevistas semiestruturadas, audiogravadas e remotas, com oito mães, recrutadas via mídias sociais. Os dados foram submetidos à análise de conteúdo indutiva.

**Resultados:**

a partir do processo analítico, foram elaboradas três categorias, com suas respectivas subcategorias: a) Redobrando os cuidados: entre o medo do imprevisível e os rituais de prevenção da contaminação; b) (Re)assumindo o controle: responsabilidades maternas sobre a desordem instaurada pela pandemia; e c) Reinventando o convívio em família: ações baseadas no cuidado apoiado.

**Considerações Finais:**

as perspectivas maternas revelam impactos multidimensionais da pandemia no cuidado infantil e evidenciam desafios e estratégias adotadas. Os achados podem subsidiar ações de suporte emocional e fortalecimento do cuidado a essas famílias.

## INTRODUÇÃO

Em 11 de março de 2020, a OMS declarou emergência de saúde pública devido ao aumento dos casos de COVID-19, levando à adoção de restrições que impactaram a população^(1)^. Algumas medidas acarretaram prejuízos importantes na economia, saúde, educação e nas relações sociais, sobretudo pela necessidade de isolamento social, mudanças de rotina, como a suspensão das atividades presenciais escolares, esportivas e de entretenimento, adoção de rigorosos protocolos de higiene e cuidados e adiamento de atendimentos e procedimentos médicos eletivos^(2)^.

No grupo de populações mais vulneráveis e afetadas durante a pandemia, estavam crianças e adolescentes, em decorrência, principalmente, da condição de fragilidade e dependência. Ademais, para além do recorte etário considerado entre 0 e 19 anos pela OMS, são pessoas em pleno processo de crescimento e desenvolvimento que merecem ter suas singularidades evidenciadas^(3,4)^. Adicionalmente, as alterações ocorridas no ambiente de aprendizagem com a transição para o ensino remoto demandaram adaptação a uma nova rotina e reduziram o convívio com seus pares e as possibilidades de atividades recreativas. Tal aspecto afetou sensivelmente a organização e o funcionamento dessas famílias^(3)^.

Nos casos em que estava associada alguma condição crônica, identificaram-se fragilidades ainda mais expostas, principalmente pela limitação dos atendimentos nos serviços de saúde e pela dificuldade de acesso à assistência^(4)^. Dentre as doenças crônicas mais comuns nessa faixa etária, está a diabetes mellitus do tipo 1 (DM1), sendo que, em 2022, constatou-se um total aproximado de 1.520.000 crianças e adolescentes menores de 20 anos com DM1 em todo o mundo. A incidência da doença vem crescendo mundialmente, com cerca 200.000 novos casos de DM1 na população infantojuvenil no ano de 2022 em relação a 2021^(5)^. Os dados brasileiros também merecem destaque, pois evidenciam cerca de 112.240 casos prevalentes de crianças e adolescentes com diabetes em 2022^(5)^.

Além de ser um importante problema de saúde pública, a DM1 é acompanhada por diversas mudanças para a população pediátrica e suas famílias, visto que, a partir do diagnóstico, devem se adaptar a novas rotinas e hábitos para um tratamento efetivo e com vistas à manutenção da qualidade de vida, tais como administração diária de insulina, controle metabólico, alimentação adequada e prática de atividade física^(6)^. Nesse contexto, torna-se importante entender como essas famílias lidam com todos esses desafios, reajustando seus papéis e desempenhando, não raro, tarefas de alta complexidade, sem treinamento e habilidades prévias^(4)^.

Trata-se de um conhecimento importante, inclusive porque as pesquisas disponíveis sobre essa temática apresentam o enfoque na saúde física e mental dessa parcela da população, o que reitera a lacuna de conhecimento a respeito dos impactos da pandemia da COVID-19 na dinâmica familiar^(7-10)^. Estudos reafirmam a necessidade de ampliar o conhecimento sobre as repercussões da COVID-19 nas famílias de crianças com doenças crônicas, abordando seus impactos a curto e longo prazo, em perspectiva complexa - ou seja, considerando as relações entre os múltiplos elementos que compõem um fenômeno e suas relações contextuais^(11-14)^. Ademais, merecem ser investigadas as características e consequências da COVID-19 em crianças e adolescentes, nos âmbitos da saúde, educação e desenvolvimento^(12,13)^.

Entendendo a família em seu sentido ampliado, como um sistema no qual os membros se influenciam mutuamente e a adaptação às adversidades ocorre de maneira dinâmica^(15)^, destaca-se o papel central das mães no cuidado de crianças com doenças crônicas. A literatura aponta que, em grande parte dos casos, são elas que assumem a responsabilidade principal pela gestão do tratamento, articulação com os serviços de saúde e suporte emocional da criança^(16)^. Nesse sentido, o fato de tal sobrecarga ter sido intensificada durante a pandemia justifica o recorte materno adotado neste estudo, a partir do qual se questiona: Quais são as repercussões da pandemia de COVID-19 no cotidiano de famílias de crianças e adolescentes brasileiros com DM1, sob a perspectiva das mães?

## OBJETIVOS

Investigar as perspectivas maternas a respeito das repercussões da pandemia de COVID-19 no cotidiano de famílias de crianças e adolescentes com diabetes *mellitus* tipo 1.

## MÉTODOS

### Aspectos éticos

Conforme diretrizes da Resolução CNS 466/2012, o estudo foi submetido e aprovado pelo Comitê de Ética em Pesquisa com Seres Humanos da instituição proponente em 2022. Após orientação clara e objetiva sobre a pesquisa, as mães que aceitaram participar assinaram o Termo de Consentimento Livre e Esclarecido (TCLE) digitalmente via Google Forms, e receberam, em seguida, uma via eletrônica do termo. Elas também poderiam solicitar aos pesquisadores uma via assinada, a qual seria enviada para seus respectivos e-mails. Durante todo o processo de coleta e análise dos dados, preservou-se o anonimato das participantes, e as entrevistas foram identificadas por números, por exemplo: E1, E2 e assim por diante.

### Tipo de estudo e referencial teórico

Para garantir o rigor deste estudo, sua descrição seguiu as orientações do *checklist Consolidated Criteria for Reporting Qualitative Research (COREQ)* ^(17)^, com apresentação detalhada do método. Realizou-se também rigorosa análise de dados por uma equipe de pesquisa experiente, o que conferiu confiabilidade e credibilidade à pesquisa. Além disso, garantiu-se a transferibilidade e confirmabilidade dos resultados por meio da apresentação da caracterização das participantes e descrição das limitações e fortalezas do estudo.

Foi desenvolvido um estudo de abordagem qualitativa, fundamentado no referencial teórico do Paradigma Complexo. Este referencial, cujo principal autor é Edgar Morin, apresenta uma visão de mundo que desafia fundamentalmente as formas de conceber a produção do conhecimento, sob pontos de vista epistemológicos, psicossocioantropológicos e éticos. A palavra complexo deriva do latim *complexus*, que significa tecido junto. Dessa forma, considera elementos contrários presentes num mesmo fenômeno, em perspectiva dialógica, acolhendo a não linearidade, a desordem e a incerteza. Busca não negar, mas superar as respostas e possibilidades do pensamento simplificador^(14)^. Traz a perspectiva sistêmica, que liga o conhecimento das partes ao todo, e a hologramática, considerando as relações entre as partes e presença do todo nestas mesmas partes. Esta escolha acolhe a visão sistêmica de família^(15)^, assumida pelas autoras, mas também considera a contextura entre as partes (criança ou adolescente e familiares), entre elas e o todo (viver com DM1 e pandemia). Entende, ainda, que os distintos elementos de determinado fenômeno permanecem em constante interação, da qual emergem propriedades singulares. Estas vivem a auto-eco-organização, ou seja, desequilíbrios e equilíbrios numa busca constante pela organização, sendo também impactada pelo ambiente. Este último aspecto denota o interesse das autoras em aprofundar a repercussões da pandemia de COVID-19 no cotidiano das famílias estudadas.

### Cenário e fonte de dados

A pesquisa foi realizada no contexto da pandemia de COVID-19, período em que medidas de distanciamento social impuseram restrições à realização de encontros presenciais. Diante disso, a coleta de dados foi conduzida remotamente, por meio de entrevistas individuais agendadas previamente e realizadas nas plataformas virtuais *Google Meet* ^®^ e *Zoom* ^®^. Participaram da pesquisa aquelas que atenderam aos critérios de seleção: mães de crianças e adolescentes entre sete e 18 anos incompletos, diagnosticados com DM1. Foram excluídas mães com menos de 18 anos de idade, bem como aquelas que, por qualquer motivo, não exerciam o papel de cuidadoras principais dos filhos no cotidiano do período pandêmico.

### Procedimentos metodológicos

Os participantes foram selecionados por amostra intencional e de conveniência, recrutados pelo método de “bola de neve” (*snowball)*. A primeira autora, enfermeira e doutoranda com experiência em estudos qualitativos, coletou os dados sob supervisão da orientadora, última autora. A pesquisadora não possuía contato prévio com as participantes. O recrutamento inicial ocorreu através da divulgação da pesquisa em redes sociais - *Instagram* e *WhatsApp*, em contas particulares dos autores, experientes nesse tipo de recrutamento. Foram postados folders contendo o tema e objetivo do estudo. Visto que a divulgação da pesquisa nas mídias sociais não continha acesso restrito, ou seja, era aberto ao público em geral, as informações de divulgação foram propagadas e alcançaram pessoas interessadas na temática. As mães que tiveram acesso a essas publicações manifestaram interesse através de contato com a pesquisadora principal via Instagram ou WhatsApp. Posteriormente, agendou-se um horário para esclarecimentos e, caso aceitassem participar, o consentimento foi obtido por assinatura do Termo de Consentimento Livre e Esclarecido. Às mães que foram incluídas, solicitou-se a indicação de outras possíveis participantes, e assim sucessivamente, seguindo o recrutamento por “bola de neve”^(18)^. A capacidade viral das redes sociais facilitou a rápida disseminação da pesquisa, ultrapassando fronteiras geográficas e conectando pessoas com interesses em comum, que, de outra forma, seriam difíceis de alcançar.

O número total de participantes foi definido a partir do momento em que os pesquisadores, através da análise progressiva e concomitante à coleta dos dados, verificaram que o conjunto de dados obtidos foi suficiente para o alcance do objetivo proposto^(19)^. A saturação teórica foi identificada quando os relatos passaram a apresentar recorrência de padrões e ausência de novas perspectivas significativas, em um sinal de que os dados coletados já contemplavam a diversidade e profundidade do fenômeno investigado. Essa decisão foi guiada por princípios fundamentais da pesquisa qualitativa, na qual a ênfase reside na qualidade e profundidade das informações coletadas, em detrimento da quantidade de participantes^(19)^. Em estudos qualitativos, a prioridade é explorar em profundidade as experiências e perspectivas dos participantes, o que, frequentemente, resulta em dados ricos e detalhados. Quando não emergiram novos temas ou perspectivas relevantes durante a análise, optou-se por encerrar a coleta de dados.

### Coleta e organização dos dados

Realizou-se uma única entrevista com cada mãe entre janeiro e março de 2023. Antes das entrevistas, a pesquisadora principal coletou os dados de caracterização, por meio de um formulário previamente elaborado pela equipe de pesquisa, o qual continha perguntas sobre idade, sexo, escolaridade, cidade de procedência da mãe e da criança ou adolescente, além da data do diagnóstico do DM1.

A entrevista semiestruturada seguiu um roteiro com questões norteadoras, a fim de explorar os seguintes aspectos: diagnóstico e cuidado da doença crônica; impacto da pandemia na rotina escolar e familiar; experiência do abandono escolar devido ao isolamento; e reinserção escolar após flexibilização das restrições. Com a delimitação oficial do término da pandemia de COVID-19 em maio de 2023 pela Organização Mundial da Saúde, a decisão de coletar dados nas proximidades desse marco temporal foi estratégica. Tal decisão visou evitar um distanciamento significativo, o que poderia levar as famílias a esquecerem aspectos relevantes, e, ao mesmo tempo, dificultar uma proximidade excessiva, capaz de suscitar emoções intensas e provocar sofrimento.

As entrevistas foram audiogravadas em aparelho de gravador de voz de forma remota e transcritas na íntegra, preservando-se o sigilo e o anonimato das participantes. Foram individuais e duraram, em média, 75 minutos, com mínimo de 30 e máximo de 165 minutos.

### Análise dos dados

A análise de conteúdo indutiva das entrevistas transcritas foi realizada em três fases: preparação (I), organização (II) e relato de resultados (III)^(20)^. (I): Na primeira fase, foram realizadas exaustivas leituras do material transcrito em busca da compreensão dos dados como um todo e identificação das unidades de significados relacionadas à temática estudada^(20)^. (II): Na segunda, houve codificação e categorização. A partir da identificação das unidades de significado, foi possível identificar os códigos que refletiram aspectos do conteúdo pesquisado, como exemplo dos códigos identificados: ansiedade e não aceitação; medo da contaminação por COVID-19; processos depressivos; sentimento de impotência da mãe; busca por ajuda profissional; apoio da família estendida, entre outros. Na sequência, agruparam-se os códigos em subcategorias e categorias, formulando-se uma descrição geral de cada uma, para apresentar de forma mais aprofundada fenômenos que respondiam ao objetivo do estudo.

As categorias foram nomeadas de acordo com as suas características e articuladas com o referencial do Paradigma Complexo, e estão apresentadas nos resultados^(20)^. Todas as fases foram realizadas pela primeira e última autora e discutidas com todos os membros da equipe de pesquisa. As pesquisadoras acumulam experiência neste tipo de análise de dados ou são especialistas no tema.

## RESULTADOS

Foram entrevistadas oito mães de crianças e adolescentes com DM1, residentes em seis cidades dos estados de São Paulo, Minas Gerais e Pará. A idade materna variou entre 33 e 50 anos, com mediana de 40 anos. No que diz respeito à escolaridade, 87,5% das participantes possuíam ensino superior completo, enquanto 12,5% tinham ensino médio.

As crianças e adolescentes com DM1 tinham entre 8 e 17 anos de idade, com mediana de 11 anos. A maioria era do sexo masculino (62,5%). O tempo de diagnóstico da DM1 variou entre 0,9 e 7 anos.

A partir do processo analítico, foram elaboradas três categorias, com suas respectivas subcategorias: a) Redobrando os cuidados: entre o medo do imprevisível e os rituais de prevenção da contaminação; b) (Re)assumindo o controle: responsabilidades maternas sobre a desordem instaurada pela pandemia; e c) Reinventando o convívio em família: ações baseadas no cuidado apoiado.

### a) Redobrando os cuidados: entre o medo do imprevisível e os rituais de prevenção da contaminação

#### Rituais de cuidado

Esta categoria trouxe aspectos relacionados a sentidos e significados percebidos pelas mães durante a pandemia e medidas preventivas, adotadas como rituais de cuidados. De acordo com as participantes, os cuidados para a prevenção da contaminação pelo coronavírus tiveram que ser redobrados para as crianças e adolescentes com DM1:

*Cuidados redobrados porque você já tem cuidado e, por ser pandemia, tinha cuidado redobrado com tudo.* (E3)

Dessa forma, os cuidados de higiene, como uso de álcool em gel, lavagem das mãos e troca de roupas ao chegar em casa demonstraram um espelho do todo pandêmico refletido nos elementos que compõem o fenômeno da rotina de cuidados ao filho com DM1:

*Eu chegava em casa, já lavava a roupa, já espirrava álcool no corpo, já lavava a mão, e ficou aquela neura, né? De limpeza, de tudo, para ele não pegar* [COVID-19]. (E1)

Neste todo repercutindo na tessitura das partes, as mães reforçaram maior necessidade de manter o isolamento social e afastar as crianças do contato com terceiros:

*Cortamos todo o convívio social, igreja, tudo. Nem supermercado ela ia; cortamos tudo mesmo.* (E7)

Para isso, utilizaram estratégias como mudanças geográficas para manter um afastamento da comunidade:

*A gente tem uma casa na represa, então, quando meu marido ficou em home office, eu falei: Vamos para lá, que lá está mais longe.* (E8)

Emergiu ainda a constante preocupação em não deixar os filhos saírem de casa:

*Nós procurávamos não sair de casa de jeito nenhum, principalmente ela.* (E7)

*Você vê que já estava introjetado nele o perigo de ter diabetes e pegar o COVID* [...] *ele: “Não, eu não quero nem sair, eu não quero ver ninguém”. Tudo por conta disso.* (E8)

#### Preocupações maternas: “o medo instaurado”

Diante do cenário pandêmico e da compreensão dos filhos como grupos de risco à COVID-19 em formas mais graves, as mães relataram um medo instaurado e constante de que seus filhos contraíssem a doença. Esse sentimento fez com que muitas se queixassem de elevada sobrecarga mental, além de sintomas depressivos, angústia, crises de choro, diminuição de apetite, perda de peso, frustração, sentimento de não aceitação e de impotência diante do cenário vivido:

*Durante a pandemia, teve frustração, teve medo, teve angústia, teve um pouquinho de desespero.* (E1)

*Eu emagreci, eu não tinha nem vontade de comer, eu ficava com aquele medo, aquele pânico na cabeça dele poder pegar* [COVID-19]. (E8)

Uma mãe afirmou temer fortemente que a produção de insulina fosse cessada no mundo, pois o foco mundial estava na produção da vacina contra o COVID-19, trazendo, explicitamente, a imprevisibilidade imposta pela pandemia:

*Nós estamos em uma situação tão difícil, todo mundo voltado para vacina. E se parar a produção de insulina, como vamos fazer? Me deu muita insegurança a questão de imprevisibilidade. Quando a gente tem um filho com doença crônica, o que você mais preza é ter o medicamento sempre à mão porque aquilo é pra vida dele, né?* (E8)

### b) (Re)assumindo o controle: responsabilidades maternas sobre a (des)ordem estabelecida pela pandemia

#### Vida pausada

Esta categoria salientou o movimento protagonizado pelas mães no qual o todo era refletido nas partes e vice-versa, ou seja, as mudanças impostas pela pandemia no cotidiano familiar e dos filhos, bem como uma busca pela (re)organização familiar frente à (des)ordem estabelecida. O momento de isolamento social e afastamento das atividades cotidianas foi por elas vivenciado como uma sensação de paralisação da vida e impossibilidade de planejamento:

*E nesse período da pandemia, onde eu parei, foi parada ali a nossa vida, parecia que não existia mais planejamento. Nós ficamos parados ali dentro de uma situação. Tínhamos que esperar o que ia acontecer.* (E2)

#### Cuidando de si: em busca de equilíbrio

As participantes relataram ainda a tentativa de controle da coordenação do cuidado, sendo protagonistas na articulação com serviços de saúde, reconhecimento de sinais e sintomas de agravamento do DM1 e organização da rotina, num movimento de auto-organização para apoiar o cuidado do filho e da família. Neste momento de sobrecarga, algumas, ao reconhecerem suas próprias fragilidades e necessidades, referiram a busca por ajuda profissional e iniciaram tratamentos farmacológicos psiquiátricos e acompanhamento em terapia:

*Eu tinha que ter um controle, eu precisava de um tratamento psicológico, eu vi que eu precisava* [...] *eu preciso demonstrar uma determinação e uma força, mas a gente não é de ferro. Eu preciso de ajuda também.* (E2)

#### Redes de apoio

Diante da situação de medo instaurado, as principais fortalezas no enfrentamento da situação foram a rede de apoio composta por profissionais de saúde que acompanhavam o cuidado de seus filhos, família estendida, grupo de mães de crianças com DM1 e a fé:

*Eu fiquei com aquele medo no começo, mas, como eu confio muito nos médicos que acompanham ele, eles me tranquilizaram muito, sabe? Então logo passou.* (E4)

*No início, quem foi fundamental pra mim foi meu pai e a esposa dele. Eles moram perto da minha casa e eles vinham todos os dias me ajudar, se não fosse eles, eu não teria dado conta porque eu chorava de desespero, de não ter capacidade de fazer tudo, dar conta de tudo.* (E3)

*Várias mães passando pela mesma situação. Isso é muito importante, uma dando apoio para a outra. Tudo o que uma descobre, através de estudo, já passa para outra também. Todo dia é conhecimento de diabetes, todo dia. Isso é muito importante.* (E6)

*Eu acredito que o que mais nos sustentou foi a fé. Foi a base de tudo.* (E2)

### c) Reinventando o convívio em família: ações baseadas no cuidado apoiado

#### Reorganização da rotina

A partir do cenário pandêmico e da instituição do isolamento social, as participantes tiveram que reorganizar as tarefas cotidianas e rotinas de cuidados com os filhos com DM1. Houve a necessidade de ajuste da alimentação por toda a família, pois as crianças passaram a ficar mais tempo em casa e a realizarem um maior número de refeições juntos:

*Eu estava aqui* [em casa] *o tempo todo com ela, sempre explicando, monitorando e a gente também mudou a alimentação por causa dela, não ficou comprando para nós e não para ela* [...] *ajudou o fato de estar em casa.* (E7)

Ao contrário do período anterior à pandemia, quando as mães não permaneciam longo período em casa, a atual circunstância, caracterizada por maior tempo de permanência domiciliar, possibilitou às cuidadoras um monitoramento mais cuidadoso da alimentação de seus filhos. Esse contexto favoreceu a adesão mais efetiva ao plano alimentar do tratamento:

*Sente muita fome, então a todo momento ele queria comer, mas, como eu estava em casa, foi mais fácil para eu controlar* [...] *eu tentava controlar um pouco, mas se a gente virar as costas, eles comem mesmo, sozinhos eles comem.* (E6)

A contagem de carboidratos também auxiliou no bom controle glicêmico neste período:

*A gente foi se virando dessa forma, né? Tentando fazer a contagem de carboidrato para não prejudicar tanto.* (E2)

*Foi até um momento que abaixou bem* [glicemia]*, durante a pandemia não teve nenhum pico alto de glicemia não.* (E1)

As crianças e os adolescentes tiveram que cessar a prática de esportes em clubes e escolas neste período. Como alternativa, as famílias passaram a realizá-las em domicílio, por meio de treinos funcionais, corrida, pular corda, jogar bola no quintal e dançar, por exemplo:

*Aí a gente fazia aqui em casa. Fazia tipo uns treinos de funcional. A gente corria aqui* [...] *às vezes ele não queria, mas era obrigado a fazer, correr na garagem, pular corda, fazer alguma coisa.* (E6)

*Ele sempre brincava muito jogando bola aqui na frente, sem se encontrar com outras crianças.* (E4)

*Por causa da pandemia, tivemos que adaptar. Ela gosta de dançar também, aí eu colocava música. Como eu estou acima do peso, eu aproveitava para dançar junto com ela, era assim.* (E7)

Outra adaptação observada nesse período foi o início dos atendimentos médicos ou nutricionais através de plataformas on-line, algo percebido por algumas mães como negativo e insuficiente:

*Daí as consultas on-line, consulta on-line não é a mesma coisa, o médico precisa ver ele. Eu nunca fiz consulta on-line com o médico dele. Eu levava. Eu fiz com a nutricionista porque eu tinha medo. Como, meu Deus? Ele não vai ver o meu filho! Como isso vai dar certo?* (E8)

#### Retomada de brincadeiras e contato próximo entre a família

Dada a necessidade de passar mais tempo em casa, as mães buscaram alternativas também para ocupar as crianças, como brincadeiras, jogos de cartas, criação de receitas alimentares e jogos de perguntas:

*Eu procurei mais esses meios de a gente fazer brincadeiras entre nós* [...] *a gente fazia brincadeira de fazer pergunta e tinha um pratinho de farinha que eu colocava na mesa e quem errasse a pergunta* [...] *é tipo torta na cara, sabe?* (E2)

Isso possibilitou que as famílias passassem mais tempo em conjunto e, dessa forma, aumentou a interação entre os membros:

*O que mais eu precisei mudar que eu observei foi a parte de interação mesmo, de lidar dentro de casa para ele não sofrer tanto com aquele distanciamento que todos estavam sofrendo.* (E2)

As crianças que tinham irmãos em casa puderem aproveitar suas companhias para brincar, jogar e assistir séries:

*A sorte é que ele tem um irmão, joga com ele, vê série, essas coisas que ajudaram.* (E8)

#### Interrupção de processos e retorno às atividades

Houve relatos de que os filhos adolescentes, em especial, vivenciaram sentimentos de ansiedade e passaram a refletir sobre o próprio diagnóstico, deparando-se com a não aceitação. O trecho a seguir ilustra a experiência de um adolescente que se viu em uma situação de interrupção de seus processos, pois iniciava um momento de troca de escola e a pandemia dificultou a construção de novas amizades, bem como a vivência de uma importante etapa de seu desenvolvimento:

*A gente percebia que ele estava ansioso. Aí, foi quando chegou o momento da não aceitação. Tipo: Eu não acredito que eu tenho diabetes!* (E1)

Neste âmbito, quando a medida de isolamento social foi sendo revista, algumas mães vivenciaram a retomada das atividades presenciais de forma dialógica - apesar do desejo de que seus filhos retornassem à escola e ao convívio social, sentiam medo e insegurança:

*Eu segurei ela um pouco, isso eu conversei com a escola porque a gente teve que reformular toda a rotina da nossa família para poder acompanhá-la no retorno à escola.* (E3)

*Eu tive que me radicalizar em um óculos rosa da certeza que ela voltaria pra casa inteira.* (E5)

## DISCUSSÃO

A pandemia de COVID-19 impactou o cotidiano de crianças e adolescentes com DM1 e suas famílias. Nesse contexto, os cuidados para evitar a contaminação foram incorporados à rotina familiar, (re)introduzindo constantemente conhecimentos e ações para o cuidado do filho. Houve a auto-eco-organização materna para retomada do controle, de modo a articular ordem e desordem impostas pelo contexto pandêmico, em busca de equilíbrio, inclusive de si mesmas, para organizar o cuidado. Tais aspectos se fazem evidentes nas categorias “(Re)assumindo o controle” e “Reinventando o convívio em família”. Tais buscas por organização se fizeram permeadas por fé, suporte de profissionais de saúde, familiares e grupos de apoio.

O cuidado de uma criança com DM1 acarreta mudanças significativas na rotina familiar e preocupações relacionadas ao manejo e tratamento desta condição^(6)^. Durante a pandemia de COVID-19, o risco de complicações mais graves em indivíduos com comorbidades agravou o sofrimento psicológico dos cuidadores de crianças e adolescentes com DM1, como evidenciado pelos relatos das mães neste estudo e confirmado na literatura científica^(9,10,21-23)^. Adicionalmente, os cuidadores que já sofriam de problemas de ansiedade e depressão experimentaram aumento deste sofrimento durante a pandemia^(24)^.

Uma das investigações que buscaram dimensionar esse impacto avaliou o impacto psicológico da pandemia em responsáveis por crianças e adolescentes com DM1, comparando-os com cuidadores de crianças sem diabetes^(25)^. Esses tinham 60% mais chances de experimentar preocupação e sobrecarga pessoal e o dobro de probabilidade de se preocupar com o cuidado dos filhos^(25)^. Inquietações semelhantes foram observadas em outros estudos, incluindo o medo de contaminação^(22)^, a preocupação com a imunidade reduzida e variações glicêmicas^(21)^, e apreensão quanto a uma possível falta de insulina e suprimentos médicos^(21,22)^. Essa apreensão exacerbada fez com que as mães desenvolvessem os rituais de cuidado apresentados nos resultados deste estudo.

Ademais, a permanência prolongada em casa alterou significativamente as rotinas diárias de crianças e adolescentes com DM1. As mães relataram maior controle sobre alimentação, monitoramento glicêmico e adesão ao tratamento, bem como necessidade de adaptar exercícios físicos para dentro de casa. Embora isso tenha intensificado a vigilância da doença, exigiu ajustes e reconfigurações na dinâmica familiar para atender às demandas das crianças e adolescentes. Esse controle foi crucial, pois, durante a pandemia, 60% das crianças e adolescentes com DM1 relataram piora na alimentação^(22)^ e 72% interromperam a prática regular de esportes^(9)^. No presente estudo, as mães não apenas buscaram controlar a alimentação, mas também se esforçaram para minimizar os efeitos negativos da interrupção das atividades físicas, incentivando a prática conjunta e diária de exercícios em casa.

A retomada do controle incluiu ações criativas e sistêmicas, como brincadeiras adaptadas, lideradas principalmente pelas mães. A dialógica autonomia-dependência marcou a retomada das atividades presenciais, em especial escolares, de crianças e adolescentes com DM1. Vislumbrou-se a tentativa de oportunizar o processo de autonomia desta população por parte das mães, ao mesmo tempo em que experienciaram temor pelos elementos imprevisíveis vividos no todo pandêmico - que trouxeram maior sensação de dependência de crianças e adolescentes pelos cuidados maternos e familiares.

A necessidade de controle interferiu no gerenciamento da doença. Durante o isolamento, houve melhora de cerca de 10% na porcentagem de tempo dentro do alvo glicêmico, além de redução significativa nos episódios de hipoglicemia e hiperglicemia em adolescentes com DM1^(26)^. Já há consenso na literatura de que a população com DM1 que permaneceu em casa durante o período de confinamento obteve benefícios com melhor controle glicêmico^(27,28)^. Esses resultados estão alinhados com nossas descobertas. Nesse sentido, evidencia-se a capacidade de autorregulação diante do caos pandêmico.

Entretanto, o exercício do controle durante a pandemia gerou contradição para as cuidadoras, pois, embora reforce a importância do cuidado parental no controle da DM1, também sobrecarrega o papel do cuidador e a convivência familiar, com consequente frustração em momentos de descontrole^(29)^. A necessidade de precisão nos cuidados gerou nos pais sentimento de culpa quando o controle do DM não atingia as expectativas^(9)^. Anteriormente, eles já enfrentavam significativo impacto emocional pelas demandas do gerenciamento da diabetes dos filhos^(30)^, e a pandemia intensificou essa carga, resultando, por vezes, em controle glicêmico adequado, porém com elevado custo emocional para os pais^(9)^. Nesse sentido, a complexidade dialoga com a necessidade de compreender a realidade de um mundo imprevisível, incluindo o planejamento de ações por profissionais de saúde que considerem esta perspectiva em futuros projetos terapêuticos - singulares e para as famílias.

A literatura já aborda a dificuldade desses pais confiarem cuidados a terceiros, como professores, o que gera carga implacável de vigilância sobre o cuidado das crianças e adolescentes com DM1^(30)^. No presente estudo, tal aspecto intensificou a insegurança em relação ao retorno às atividades escolares presenciais. A confiança depositada na equipe de saúde e nos familiares, por outro lado, desempenha papel fundamental na manutenção do controle e no estabelecimento de uma rotina familiar consistente^(23,29)^. Durante a pandemia de COVID-19, o suporte social e a telemedicina foram fundamentais para redução do sofrimento psicológico vivenciado pelas famílias^(24)^, apesar da necessidade de adaptações para garantir o cuidado contínuo, como a aquisição de habilidades no uso de tecnologias^(23)^. No entanto, a dificuldade em aceitar a eficácia das consultas on-line foi relatada pelas mães neste estudo e reforçada por outros cuidadores, os quais expressaram preferência por atendimentos presenciais^(9)^.

A complexidade evidenciada pela instabilidade emocional e por mudanças abruptas na rotina pode dificultar a percepção de fatores de enfrentamento e progressos positivos^(15)^. Nesse sentido, torna-se essencial cuidar também do cuidador. A longa duração das entrevistas realizadas neste estudo ressalta a necessidade de fornecer espaço para as mães compartilharem suas experiências e preocupações. A variedade de relatos destaca a importância de explorar a fundo essas experiências em pesquisas futuras. Compreender desafios e necessidades é essencial para fornecer suporte psicossocial e recursos de autocuidado. Ao acolher e apoiar as mães nesse contexto, é possível promover bem-estar, inclusive para as crianças com DM1, contribuindo para ganhos na qualidade de vida e melhores resultados de saúde. Apesar da disponibilidade de numerosos estudos na literatura científica sobre as consequências da pandemia, ainda há lacuna de pesquisa para explorar as lições aprendidas com os cuidados prestados a crianças e adolescentes com doenças crônicas durante esse período desafiador.

### Limitações do estudo

As fortalezas deste estudo incluem a coleta de dados após o isolamento social, o que foi vantajoso por possibilitar o compartilhamento de experiências das mães acerca do retorno às atividades. O uso de ferramentas tecnológicas para coleta de dados qualitativa de maneira remota propiciou acesso a participantes de diferentes localidades. Ademais, a condução de um estudo qualitativo, seguindo as orientações do *checklist* COREQ, garantiu rigor na descrição dos resultados.

Uma possível limitação do estudo é o nível de instrução das mães entrevistadas, todas com graduação (n=7) ou cursando (n=1), o que não reflete a realidade de todas as famílias de crianças e adolescentes com DM1, pois sabe-se que a pandemia afetou de forma mais profunda famílias em situações de vulnerabilidade^(24,31)^. Além disso, a coleta de dados remota, embora vantajosa, limita o acesso a populações economicamente desfavorecidas, cujos impactos podem ter sido distintos. As evidências apresentadas refletem as particularidades vivenciadas pelas famílias de crianças e adolescentes com DM1 incluídas neste estudo. O processo de transferibilidade e interpretação dos resultados para outros contextos, como outras doenças crônicas infantis, deve ser realizado com cautela, considerando todas as características contextuais dos participantes deste estudo.

### Contribuições para a área da enfermagem, saúde ou política pública

A perspectiva materna, na visão qualitativa, traz uma contribuição nova para a literatura e prática clínica, focada na experiência e na complexidade experienciada, dado que os principais estudos publicados sobre as repercussões da pandemia de COVID-19 são quantitativos e, portanto, contribuem apenas com dados numéricos, em sua maioria a respeito do controle glicêmico da população infantojuvenil com DM1. Considera-se essencial reconhecer e compreender como a sobrecarga materna foi ampliada durante a pandemia, para que os profissionais de saúde possam desenvolver recursos de apoio emocional aos cuidadores e oferecer suporte no cuidado das crianças e adolescentes, especialmente em momentos de elevado estresse, como períodos pandêmicos e pós-pandêmicos. Nesse sentido, destaca-se a importância da enfermagem no acompanhamento longitudinal de crianças e adolescentes com DM1, não apenas no monitoramento clínico e controle glicêmico, mas também no acolhimento, na escuta qualificada e na orientação contínua às famílias. Isso pode promover uma melhor coordenação do cuidado e maior qualidade de vida para as famílias que convivem com a DM1 na infância e na adolescência.

## CONSIDERAÇÕES FINAIS

Este estudo investigou as perspectivas maternas sobre as repercussões da pandemia de COVID-19 no cotidiano de famílias de crianças e adolescentes com DM1. Diante da (des)ordem instaurada pelo contexto pandêmico, medidas preventivas e mudanças de rotina foram incorporadas ao cotidiano para proteção contra o vírus e prevenção de complicações pela DM1. A busca pela (re)organização familiar, sobretudo pelo retorno a atividades após o isolamento social, reintroduziu conhecimentos e ações criativas para o cuidado do filho.

## Data Availability

Os dados de pesquisa estão disponíveis no corpo do artigo.
